# Rat bite fever caused by *Streptobacillus moniliformis* infection in a Chinese patient

**DOI:** 10.1186/s12879-019-4281-z

**Published:** 2019-07-17

**Authors:** Wei-Wei Zhang, Yi-Bing Hu, Guo-Xin He, Yu Zhou, Liang Hong, Ji-Guang Ding

**Affiliations:** 1 Department of Infectious Diseases, The Third Affiliated Hospital, Wenzhou Medical University, Wenzhou, Zhejiang, 325200 People’s Republic of China; 2 Department of Gastroenterology and Hepatology, Jinhua Municipal Central Hospital, Jinhua Hospital of Zhejiang University, Jinhua, 321000 Zhejiang, People’s Republic of China

**Keywords:** *Streptobacillus moniliformis*, Rat bite fever, Meta-next generation sequencing

## Abstract

**Background:**

Rat bite fever (RBF), a severe infectious disease, can result from transmission of the pathogen *Streptobacillus moniliformis* (*S. moniliformis*) by rat bite. RBF diagnosis can be overlooked.

**Case presentation:**

We present a case of RBF in a Chinese patient who was infected with *S. moniliformis* in mainland China. Meta-next generation sequencing (mNGS) was used to identify potential pathogens and detected *S. moniliformis* genome sequences in the pustular sample in less than 72 h. Then the diagnosis was validated by polymerase chain reaction analysis. Despite having severe RBF with complications, this 54-year-old male patient was successfully cured with penicillin as a result of timely pathogen-based diagnosis.

**Conclusions:**

Physicians should inquire about recent rat exposure and consider the possibility of RBF when a patient develops unexplained fever and rashes. mNGS is a new diagnostic technology and may identify RBF pathogens even when blood culture results are negative.

## Background

Rat bite fever (RBF) is a severe infectious disease, resulting from transmission of pathogen by rat bite, and *Streptobacillus moniliformis* (*S. moniliformis*) is a known pathogen for RBF [1]. The mortality or RBF has been reported to be as high as 13% in the absence of appropriate and timely treatment [2]. *S. moniliformis-*infected RBF cases are mostly reported in the western hemisphere, and only rarely reported in Asia [3]. Herein, we present a case of 54-year-old man with severe RBF disease caused by *S. moniliformis*, which was identified by meta-next generation sequencing (mNGS) and validated by polymerase chain reaction (PCR) analysis. To the best of our knowledge, this is the first documented RBF case infected with *S. moniliformis* in mainland China. This report discusses the clinical course and management in this case as well as reviews the literature related to RBF.

## Case presentation

A 54-year-old man was admitted to the emergency room (ER) of the Third Affiliated Hospital, Wenzhou Medical University on 20 July 2018. He had a 4-day history of chills, fever (39.0 °C), malaise, fatigue, myalgia and mild diarrhea, and had been treated with herbal medications for 2 days. His fever had been brought down; however, his fatigue and myalgia were exacerbated. He developed a yellowish complexion on the day prior to presenting at our ER.

**Fig. 1:**
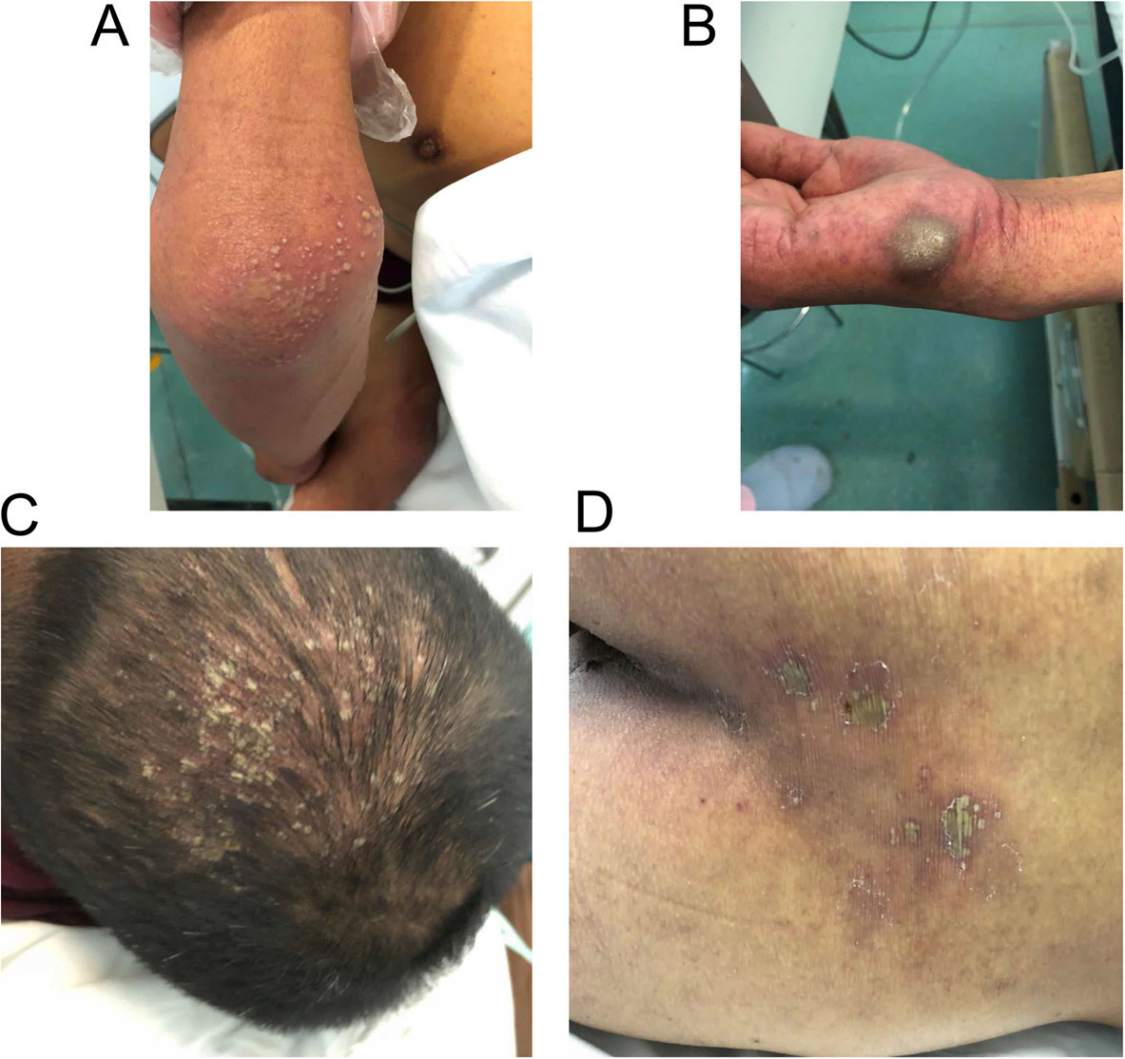
Skin manifestations of RBF on the right elbow (**a**), right palm (**b**), head (**c**) and hip (**d**) upon admission. Dusky-purple pustular and petechial lesions are clearly visible

Upon admission, the patient had a normal temperature of 36.5 °C, heart rate of 95 beats/min, blood pressure of 96/77 mmHg, respiratory rate of 18 breaths/min and oxygen saturation of 100% in room air. His Glasgow Coma Scale score was 13. He also had cutaneous and scleral icterus. The patient’s urine volume of 24 h was 210 ml. The most prominent appearance of his skin was numerous scattered rashes. Many dusky-purple pustular and petechial lesions appeared on his head, right elbow, right palm, hip and feet (Fig. 1). No bite wound was seen. He was conscious with normal cardiac, pulmonary, abdominal and other physical findings.

In discussing recent events leading to his current conditions, he recalled being bitten on his right foot by a wild rat in his house 1 week prior to the onset of symptoms. He was alerted by the rat bite but did not experience any distress. The bite wound healed normally.

Computed tomography (CT) imaging of the head, chest and abdomen was unremarkable except for bronchiectasis in the right lung. Laboratory analyses found an elevated C-reactive protein (CRP) level of 225 mg/L, a white blood cell (WBC) count of 5.6 × 10^9^/L with 89.8% neutrophils and a reduced platelet (PLT) count of 4 × 10^9^/L. Additional findings included: procalcitonin level > 100 ng/mL, serum total bilirubin (TB) of 501 μmol/L, serum creatine (CR) concentration of 764 μmol/L, hemoglobin level of 171 g/L, serum albumin (Alb) concentration of 31 g/L, and alanine aminotransferase (ALT) level of 93 U/L.

**Fig. 2:**
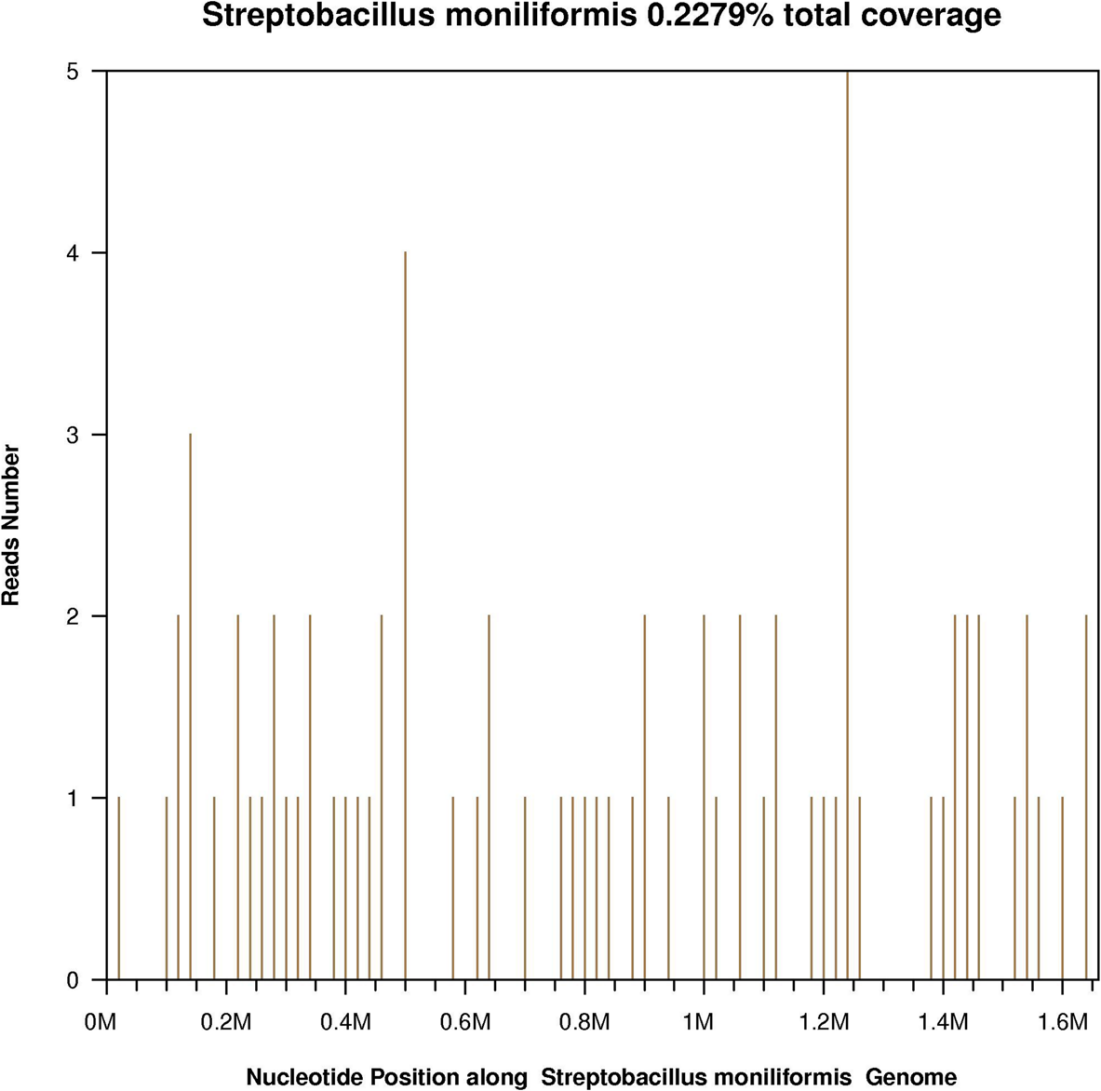
Sequence reads mapped to *S. moniliformis* by mNGS data. A total of 86 reads mapped to *S. moniliformis* in the reference database, which contains about 8000 pathogen genomes, corresponding to a total coverage of 0.2279%

The initial diagnosis included hemorrhagic fever with renal syndrome (HFRS), sepsis, kidney dysfunction and liver dysfunction. Because of the disease severity, the patient was transferred to our intensive care unit, and a blood sample was sent for bacterial culture. In the meantime, empirical treatment with an intravenous drip of tazobactam/piperacillin (4.5 g every 8 h) was initiated. Continuous renal replacement therapy (CRRT) was applied to treat renal failure. On day 4, blood culture yielded a negative result. However, the WBC count was further elevated to 13.1 × 10^9^/L with 11.3 × 10^9^/L neutrophils accompanied by re-emergent fever (peak 38.9 °C), and the CRP and Cr concentrations remained at high levels. Hepatic function continued to deteriorate (ALT from 93 U/L to 258 U/L in 6 days). Oral doxycycline (0.1 g every 12 h) was then added to the antibiotic regimen to broaden the antibacterial spectrum. No therapeutic response was observed. Considering blood culture had failed to reveal a pathogen, a pustular sample from his right ankle was collected and sent for unbiased meta-next generation sequencing (mNGS) (BGI, Shenzhen, China), in which the pool of detected sequences can match a sequence database of 8000 pathogens including bacteria, fungi, virus and parasite. Within 72 h, mNGS detected 86 of 20 million reads that matched *S. moniliformis* (Fig. 2). To confirm infection by this rare pathogen, a specific PCR was performed using the same pustular sample. The resultant PCR product was confirmed by Sanger sequencing. The PCR primers were S5 (CATACTCGGAATAAGATGG) and AS2 (GCTTAGCTCCTCTTTGTAC), which target a *S. moniliformis* specific region of 16S rRNA [4]. The empirical tazobactam/piperacillin treatment was immediately replaced with penicillin (800,000 IU intravenously every 8 h) for 14 days. The patient’s clinical symptoms were improved after penicillin treatment. The skin pustular lesions erupted, then shrank and scabbed. His WBC count, CRP level, PLT count and serum Cr level returned to normal. The patient made a complete recovery during a follow-up of 3 months after discharge.

## Discussion and conclusions

RBF was first described in the United States in 1839, and two outbreaks were reported in the US in the early 1990s [5]. The youngest reported patient was 2 months old [6]. RBF is usually transmitted to humans by rat bite, but also can be transmitted by ingestion of contaminated water or food [7]. Animals like cats, dogs and pigs can also be at risk for RBF [8, 9]. No case of RBF transmission from human to human has been reported.

RBF manifestations include rash (61%), fever (92%), headache (34%), vomiting (40%) and polyarthralgia (66%), none of which is specific [10]. Wang et al suggested that septic arthritis that may accompany RBF is unique and may be considered a separate entity [11]. The RBF prognosis is favorable if effective antibiotic treatment is timely initiated. Conversely, severe complications can follow, resulting in a mortality rate of 7–13% [3]. Recently, Eisenberg et al reported acute tetraplegia in a snake keeper in relation to RBF. The patient’s condition was so bad that he was sedated, mechanically ventilated and even admitted to the intensive care unit [12]. Endocarditis is the primary cause of death, accounting for up to 53% of cases of morality [9, 13, 14].

RBF can be caused by either *S. moniliformis* or *Sprillum minus* [15]. *S. moniliformis*, a nonmotile Gram-negative rod, is often detected in North America but relatively rare in Asia. With the increasing popularity of rats as pets, pet vendors and buyers are at an increased, unaware risk for *S. moniliformis* infection [16]. Some studies showed that nearly 10% of rat bites lead to *S. moniliformis* infection [5], and the patient in this case was bitten by a rat a week before admission. In 2012, Chean et al reported a case of RBF with *S. moniliformis* infection in an HIV-infected patient [17]. The present case represents the first documented RBF case with *S. moniliformis* infection in mainland China.

The pathogenesis of RBF caused by *S. moniliformis* is unclear. Biopsy of skin lesions in a previous RBF patient revealed leukocytoclastic vasculitis, and autopsy has found hepatosplenomegaly, degenerative alterations in liver and kidneys, erythrophagocytosis and lymph node sinus hyperplasia in reported RBF patients [3].

Because RBF manifestations resemble those of other diseases including hemolytic uremic syndrome, Lyme disease, rheumatoid arthritis, and postinfectious arthritis, it is critical to make early attempts to identify possible pathogens [11, 18]. However, *S. moniliformis* isolation requires specific culture medium that is enriched with 10–30% blood or serum. The negative culture results obtained in the present case suggest the low sensitivity of non-blood enriched culture medium.

Several other assays are more sensitive than culture for *S. moniliformis* detection, including gas-liquid chromatography, PCR and 16S-rRNA sequencing [4]. Previously, PCR was used to amplify *S. moniliformis* genome sequences from the bite site [19], and most cases were identified by 16S-rRNA sequencing. Currently, high-throughput sequencing techniques like mNGS are becoming increasingly important for the detection of rare infections, as shown by this case. This advanced technology will enable timely diagnosis and treatment of RBF and is expected to result in excellent outcomes, as experienced in this case. We identified the pathogen in the present case using two molecular methods but from the same pustular sample, which may be a limitation in this case. Testing of a sample from the rat for verification would have been good as well but was not possible. Even still, in future cases, we will performing sequencing analyses using samples from multiple pustule for improved diagnostic accuracy. Furthermore, using culture or histopathological examinations to verify the results of mGNS may increase the specificity. However, such techniques could produce false negativity at the same time, which may delay the administration of effective antibiotics, as was critical in this case. Overall, we are confident about the diagnosis in the present case and publish it expecting this report to broaden the discussion on using mGNS for identifying clinical pathogens and ot help establish standardized requirements when a pathogen diagnosis is based on mGNS technology.

Penicillin G is the first choice of antibiotics for RBF treatment and a 7–14-day course (400,000–600,000 IU/day) is recommended for adults in the absence of complications. If no response is observed in 2 days, the dose can be increased to 1.2 million IU per day [20]. In the present case, the dose of penicillin was increased in view of the severe complications. Doxycycline (100 mg bid) can be used in penicillin-allergic patients [2]. *S. moniliformis* is also sensitive to Clindamycin, erythromycin and ceftriaxone, although the standard treatment durations need to be established [3]. Several disinfection measures are recommended to prevent RBF by the USA Center for Disease Control and Prevention (CDC), including washing hands with disinfectants, wearing protective gloves and avoiding close contract with rats [21].

In conclusion, we described the first documented case of RBF resulting from infection with *S. moniliformis* in mainland China. Blood culture in this case was negative, but after the pathogen was identified by mNGS and confirmed by PCR analysis, the patient was successfully treated with penicillin. Physicians should inquire about recent rat exposure and consider the possibility of RBF when a patient develops unexplained fever and rashes. mNGS is a new diagnostic technology and may identify RBF pathogens even when blood culture results are negative.

## Data Availability

All data generated or analyzed during this study are included in this published article.

## Abbreviations

ALT: Alanine aminotransferase
CR: Creatine
CRP: C-reactive protein
CRRT: Continuous renal replacement therapy
ER: Emergency room
HFRS: Hemorrhagic fever with renal syndrome
HIV: Human immunodeficiency virus
mNGS: Meta-next generation sequencing
PCR: Polymerase chain reaction
PLT: Platelet
RBF: Rat bite fever
*S. moniliformis*: *Streptobacillus* moniliformis
TB: Total bilirubin
WBC: White blood cell
